# Carnivora Population Dynamics Are as Slow and as Fast as Those of Other Mammals: Implications for Their Conservation

**DOI:** 10.1371/journal.pone.0070354

**Published:** 2013-08-12

**Authors:** Madelon van de Kerk, Hans de Kroon, Dalia A. Conde, Eelke Jongejans

**Affiliations:** 1 Radboud University Nijmegen, Institute for Water and Wetlands Research, Department of Experimental Plant Ecology, Nijmegen, The Netherlands; 2 Department of Wildlife Ecology and Conservation, University of Florida, Gainesville, Florida, United States of America; 3 Max Planck Odense Center of Evolutionary Demography, Institute of Biology, University of Southern Denmark, Odense, Denmark; 4 Centre for Research and Conservation, Royal Zoological Society of Antwerp, Antwerp, Belgium; 5 Radboud University Nijmegen, Institute for Water and Wetlands Research, Department of Animal Ecology and Ecophysiology, Nijmegen, The Netherlands; University of Toronto, Canada

## Abstract

Of the 285 species of Carnivora 71 are threatened, while many of these species fulfill important ecological roles in their ecosystems as top or meso-predators. Population transition matrices make it possible to study how age-specific survival and fecundity affect population growth, extinction risks, and responses to management strategies. Here we review 38 matrix models from 35 studies on 27 Carnivora taxa, covering 11% of the threatened Carnivora species. We show that the elasticity patterns (i.e. distribution over fecundity, juvenile survival and adult survival) in Carnivora cover the same range in triangular elasticity plots as those of other mammal species, despite the specific place of Carnivora in the food chain. Furthermore, reproductive loop elasticity analysis shows that the studied species spread out evenly over a slow-fast continuum, but also quantifies the large variation in the duration of important life cycles and their contributions to population growth rate.

These general elasticity patterns among species, and their correlation with simple life history characteristics like body mass, age of first reproduction and life span, enables the extrapolation of population dynamical properties to unstudied species. With several examples we discuss how this slow-fast continuum, and related patterns of variation in reproductive loop elasticity, can be used in the formulation of tentative management plans for threatened species that cannot wait for the results of thorough demographic studies. We argue, however, that such management programs should explicitly include a plan for learning about the key demographic rates and how these are affected by environmental drivers and threats.

## Introduction

Carnivora is a highly threatened order with a quarter of its species being in the Red List categories of Vulnerable, Endangered or Critically Endangered and with five species already listed as Extinct [1]. Carnivorous species are among the most threatened mammals [2], [3]. Because species of Carnivora exert an important ecological role in their communities, either as top or mesopredators [4], it is essential to manage their populations if we aim to conserve ecosystems and slow down the current extinction trends. Extinction is ultimately a demographic process; the result of changes in mortality and fertility that lead to a negative population growth. Therefore demographic data are essential for the development of population management programs. However, the lack of data for most threatened species makes population analyses and forecasting unreliable [5]. Reliable demographic data are particularly hard to obtain for Carnivora due to their tendency to be elusive, nocturnal and occasionally dangerous [6]. In this paper we analyze if generalizations can be made among Carnivora to inform demographic models for population management of species for which no data are available.

Carnivora species often fit all the labels of conservation urgency. They are both indicator species, (i.e. their occurrence being an indicator for the ‘health’ of the ecosystem), and keystone species; some are at the top of the food chain [7] and others play an important role as mesopredators in their ecosystems [4]. Especially large Carnivora species play an important role since they shape prey communities [8]. Also, they serve as umbrella species for many other species; protecting carnivores generally requires protecting entire ecosystems. Carnivores are also very appealing to the public, usually ranking in the top of popular animals, serving as flagship species. And, maybe most importantly, many of the characteristics that are generally used to describe vulnerable species apply to many Carnivora [9]. This vulnerability includes a narrow geographical range, in many cases large home ranges, low population densities, specialized niche requirements, and being hunted by humans. Many conservation activities are meant to increase populations of a certain species because of its rarity and extinction risk [10]. However, rarity provides an additional challenge since studying large numbers of individuals of rare species is difficult while long periods of extensive monitoring are required to obtain sound data. Moreover, monitoring and research activities such as radio tracking require expensive equipment or even the use of helicopters. These factors together make it extremely difficult to gather long-term datasets of Carnivora populations.

Transition matrix models provide a transparent way to model the population dynamics of a species, and to project the growth rate and extinction risk of a population [11],[12],[13]. Using matrix models, it is feasible to identify which critical phases in the life cycle should be targeted by management strategies [12],[14]. This is possible by assessing the elasticity values in the matrix, quantifying how the population growth rate is affected if a perturbation occurs on a particular vital rate (e.g. juvenile survival or adult survival). For example, by estimating the elasticity patterns of sea turtles, Crowder et al. [15] found that a management intervention will have more effect if it is directed towards survival rather than fertility.

However, for many species sufficient demographic data to develop a model are not available, take too long or are too expensive to collect. To inform management about the population dynamical responses of such species it would be very useful if elasticity values of life cycle components could be estimated from simple life history characteristics. Comparisons of multiple species can reveal general relationships between elasticity values and species characteristics, as previously been done for plants [16],[17], birds [18],[19], turtles [20],[21] and mammals [22].

Animal life histories are generally classified along a ‘slow-fast continuum’ [18],[23],[24]. Slow animals mature and reproduce late, live long, and produce few offspring. Fast animals start reproducing early, die young, and generally produce large litters. Heppell et al. [22], comparing elasticity values across 50 mammals, suggested that management strategies towards either increasing or reducing population growth rates should target offspring survival for ‘fast’ mammals, and adult or juvenile survival rates for ‘slow’ mammals. However, it is important to assess if these generalizations can be applied particularly to the order Carnivora. Although Carnivora species expand through the fast and slow continuum, the fact that they have a specific place in the food chain, as top or mesopredators, means that population densities are usually low, causing them to be more vulnerable for known threats. Moreover, Carnivora shape other Carnivora population dynamics. For example the removal of one Carnivora species can profoundly affect the density of other Carnivora [25]. Interactions between Carnivora are likely to differ from interactions among other groups, since they can often result in the death of one of the individuals involved (referred as interference intraguild interactions, reviewed by Linnell and Strand [26]). These particular characteristics of Carnivora as apex or mesopredators and the interference intraguild interactions among them, could be reflected in different elasticity patterns from other mammals. The aim of this paper is therefore to investigate if there are rules of thumb for elasticity patterns in Carnivora across the slow and fast continuum and compare them to those described for mammals in general [22]. If elasticity patterns of Carnivora are similar to the ones of other mammals this will facilitate to inform management programs, for one of the most threatened groups of mammals.

### Population attributes specific to Carnivora

The order of the Carnivora is a well-defined taxon representing a wide range of life histories [7]. It contains ca. 285 species of placental mammals, and includes many carnivorous species such as canids and felids, but also omnivores, such as the black bear, and a few herbivores, like the giant panda. Other life history aspects are diverse as well: the Carnivora include both the stoat (body mass 140 g) and the walrus (1500 kg), both the cheetah (savannah habitat) and the sea otter (oceanic), both the red fox (home range 0.20 km^2^) and the African wild dog (2000 km^2^), and both the island fox (forming monogamous pairs) and the grey wolf (living in social groups). Beside this large trait diversity, the conservation status of Carnivora is also highly variable. Some species, such as raccoons, are considered pests, while extinction seems inevitable in the wild for some others, such as the giant panda [27]. Many Carnivora live in complex social groups, and show coordinated behavior within these groups, such as cooperative hunting. Species living in social groups are more complicated to model, because their group composition influences vital rates such as survival and fecundity. Grey wolves for example experience a much higher mortality when living as individuals than when living in a group, and only the dominant male and female in a group generally reproduce [28].

Perhaps the most conspicuous fact about Carnivora is that most of them are hunters. Therefore, their prey also regulates the dynamics of Carnivora populations. These predator-prey dynamics may be counterintuitive, because an increase in prey density can sometimes increase the effect of competition among carnivores, instead of weakening it [29]. More than other taxa, many Carnivora species have difficulties living alongside each other and people. They often cause property damage, and large carnivores kill cattle and could even kill people. Large carnivores require vast areas to survive, and they compete with each other for prey and hunting territories, and they compete with people for game, space and resources [30]. In almost every large carnivore population, people are responsible for most mortality [30]. Additionally, within carnivore guilds it is common to have complex interactions such as exploitative completion for resources and interspecific interference interactions [31]. Both types of interactions have as a result that population changes of one Carnivora species could lead to mesopredator-release or suppression [26]. These population attributes specific to Carnivora complicate the modeling of their population dynamics as well as their conservation.

## Methods

We performed a literature search in Thomson's on-line Web of Science database and in Google Scholar using the strings or search terms: “population dynamics” or “demography” and “matrix” or “elasticity”. Articles about non-Carnivora species were discarded. We reviewed the title and abstract of the remaining articles, along with the full text if necessary, and we added some articles found through cross-referencing. Only articles were selected that 1) comprised a wild Carnivora population, and 2) used matrix modeling, and 3) provided the transition matrix or allowed for reconstruction of the matrix by showing the used data.

We found a total of 35 studies about wild Carnivora populations, comprising 27 taxa (Table 1), and reconstructed 38 matrices used in these studies. If a study presented multiple matrices for different scenarios, only the matrix for the average scenario was used. If a study presented different matrices for consecutive years, the average matrix was used. We calculated the population growth rate λ, elasticity matrices and generation time (defined here as log(R_0_)/log(λ), where R_0_ is the net reproductive rate) using the *popbio* package in R [32]. We made all data and parameters obtained available in the COMADRE database of the Max Planck Institute for Demographic Research, which will be available online in July 2013.

**Table 1:** The Carnivora species included in this paper.

Family	Species	IUCN status^a^	Body mass	Life span	Mat dim^b^	R1^c^	λ^d^	Gen. time^e^	Age 50% elas	Reference
Canidae	Grey Wolf *(Canis lupus)*	LC	38	10	6	2	1.33	6.2	5.1	[28]
	Grey Wolf *(Canis lupus)*	LC	38	10	10	2	1.35	4.3	3.7	[45]
	African Wild Dog *(Lycaon pictus)*	E	36	10	3	2	1.29	5.5	4.3	[66]
	Culpeo Fox *(Pseudalopex culpaeus)*	LC	12	6	3	1	1.29	4.1	3.4	[67]
	Island Fox *(Urocyon littoralis)*	CE	2.8	7	2	0	0.87	4.4	5.3	[36]
	Island Fox *(Urocyon littoralis)*	CE	2.8	7	3	1	0.64	3.7	4.8	[50]
	Red Fox Urban 1 *(Vulpes vulpes)*	LC	11	5	6	0	1.03	3.9	1.6	[39]
	Red Fox Urban 2 *(Vulpes vulpes)*	LC	11	6	6	0	1.08	3.9	1.8	[39]
	Red Fox Rural 1 *(Vulpes vulpes)*	LC	11	6	5	0	1.06	3.8	3.0	[39]
	Red Fox Rural 2 *(Vulpes vulpes)*	LC	11	6	6	0	0.95	4.0	2.8	[39]
Felidae	Cheetah *(Acinonyx jubatus)*	V	65	12	8	2	0.96	15	21	[68]
	Ocelot *(Leopardus pardalis)*	LC	35	11	4	3	1.05	9.7	9.5	[69]
	Leopard *(Panthera pardus)*	NT	60	15	4	3	1.09	9.0	8.1	[70]
	Cougar *(Puma concolor)*	LC	48	12	12	2	0.92	4.9	6.2	[41]
	Cougar *(Puma concolor)*	LC	48	12	5	2	0.88	6.7	7.6	[46]
	Florida panther *(Puma concolor coryi)*	LC	73	18	19	2	1.06	5.0	5.2	[71]
Mustelidae	Eurasian Otter *(Lutra lutra)*	NT	7	16	2	1	1.26	4.4	3.8	[72]
	Sea Otter *(Enhydra lutris)*	E	33	20	20	3	1.13	9.0	8.9	[73]
	River Otter *(Lontra canadensis)*	LC	8	15	3	1	1.02	5.8	6.2	[74]
	Badger *(Meles meles)*	LC	13	15	15	2	0.99	5.8	7.3	[75]
	Stoat *(Mustela erminea)*	LC	0.14	4	3	0	1.26	5.8	1.5	[42]
Odobenidae	Walrus *(Odobenus rosmarus)*	–	1500	25	26	6	0.98	34	63	[76]
Otariidae	Steller Sea Lion *(Eumetopias jubatus)*	NT	300	31	32	3	1.00	10	11	[77]
	Steller Sea Lion *(Eumetopias jubatus)*	NT	300	31	14	3	1.01	13	14	[78]
	New Zealand Sea Lion *(Phocarctos hookeri)*	V	160	25	26	4	1.00	10	12	[79]
	California sea lion *(Zalophus californianus)*	LC	100	20	3	1	0.95	7.6	9.2	[40]
Phocidae	Grey Seal *(Halichoerus grypus)*	LC	150	25	7	5	1.08	16	14	[43]
Ursidae	Giant Panda *(Ailuropoda melanoleuca)*	E	100	25	13	6	1.00	12	12	[38]
	Black Bear *(Ursus americanus)*	LC	180	24	69	2	1.02	9.2	11	[37]
	Black Bear *(Ursus americanus)*	LC	180	24	5	5	0.95	11	14	[54]
	Black Bear *(Ursus americanus)*	LC	180	24	4	4	0.78	6.1	7.2	[80]
	Florida Black Bear *(Ursus americanus floridanus)*	LC	82	10	5	2	1.01	13	17	[81]
	Eurasian Brown Bear *(Ursus arctos arctos)*	LC	150	20	4	4	1.19	10	7.6	[82]
	Grizzly Bear *(Ursus arctos horribilis)*	LC	160	20	50	3	1.05	9.6	10	[49]
	Grizzly Bear *(Ursus arctos horribilis)*	LC	160	20	10	4	1.01	9.2	10	[47]
	Grizzly Bear *(Ursus arctos horribilis)*	LC	160	20	21	5	1.03	12	12	[48]
	Japanese Brown Bear *(Ursus arctos yesoensis)*	LC	190	25	5	5	1.06	17	16	[83]
	Polar bear *(Ursus maritimus)*	V	680	25	6	6	0.99	19	26	[84]

a) LC = Least Concern, V = Vulnerable, NT = Near Threatened, E = Endangered, CE = Critically Endangered.

b) Matrix dimensions.

c) Age of first reproduction according to matrix.

d) Projected population growth rate.

e) Generation time (log(R_0_)/log(λ)).

### Elasticity as a tool for conservation

Perturbation analyses are a tool to determine the importance of transitions in a transition matrix for the population growth rate (λ). Most commonly used for questions of species conservation are elasticities or proportional sensitivities, given by *e_ij_* = (*a_ij_*/λ) (∂λ/∂*a_ij_*), with matrix element *a_ij_* as the transition from stage *j* to stage *i* in the transition matrix [14],[33]. The λ-elasticities of all matrix elements sum up to one, and quantify the relative contributions of the matrix transitions to λ. High elasticity values indicate on which transitions in the life cycle population growth relies most. If interpreted with care (e.g. in conjunction with actual λ values), elasticity values indicate targets for conservation [12],[14].

The λ-elasticity values of the matrix elements were summarized into three groups, namely juvenile survival (*Sj*), adult survival (*Sa*) and fecundity (*F*). All individuals that are able, or have been able, to reproduce were classified as adults, and all younger individuals were referred to as juveniles. The elasticity values of *Sj*, *Sa* and *F* sum to one and were plotted in a triangular graph (see also [22]), giving information on the distribution of the elasticity values of *Sj*, *Sa* and *F*, compared among species.

Since the distinction between juveniles and adults might not always be strict (sometimes only part of the animals of a certain age become reproductive adults, while others wait until later years), we also investigated the λ-elasticity values of life cycle loops [34]. Reproductive loops in the life cycle are the actual pathways that individuals follow from birth to reproduction and offspring formation. Each transition matrix can be decomposed into a finite number of closed loops, the elasticities of which also sum to one as with regular elasticity analysis [34],[35]. Loop analysis is particularly useful for comparison of the relative importance of reproductive loops of various duration for population growth. In an age-based model, loops differ by the age of reproduction. One way to quantify the relative position of a species on the slow-fast continuum is by arranging loops from short to long and quantifying the age (i.e. loop length) by which the cumulative loop elasticity reaches 50% of total elasticity. To allow this analysis for all species, all transition matrices must be age-based (Leslie matrices) and we have turned transition matrices that were partly stage-based into age-based matrix models (see Appendix S1 for more details). The resulting age-based models had exactly the same, or very comparable, λ values as the original stage-based models. In the cases of simple Leslie matrices it was easy to distinguish the loops, but in more complex cases we applied the loop detection algorithm of Güneralp [35], starting with shorter loops as further explained in Appendix S1.

Finally, we investigated the relationships of these elasticity distributions (of *Sj*, *Sa* and *F*, as well as the age associated with a cumulative 50% loop elasticity) to several life history traits and matrix model characteristics, to detect which traits are most strongly related to life history strategies and can be used as predictors of population dynamics.

## Results

### Carnivora matrix models in the literature

The size of the Carnivora transition matrices in the literature ranged from 2×2 [36] to 69×69 [37] with an average of 12×12 and a median of 6×6. More than half [24] of the 35 studies used elasticity analyses. Only three studies explicitly modeled populations from different locations [38],[39],[40], but 27 papers incorporated temporal variation.

Many authors struggled to obtain sufficient demographic data for constructing a matrix model. Some species occurred in such low densities that even sampling the whole population was not sufficient to get reliable estimates for all parameters (e.g. for cougars [41]). Another study used an impressive 23 papers to estimate the parameters of their black bear model [37].

Most studies aimed to provide information that can be used to optimize conservation management and many authors discussed management implications in a separate section. Some of them (13 studies) argued which transition should be targeted with conservation efforts, some explored the possible effects of different management strategies (3 studies), and others (11 studies) provided a population viability analysis (PVA), estimating extinction risks. Some studies focused on other applications, such as pest management (e.g. for stoats [42]) or assessing the possibility of sustainable harvest (e.g. of grey seals [43]). From the literature we finally analyzed a total of 27 taxa, which accounts for 9% of the 285 carnivores. Those include 11% of the threatened Carnivora species within Red List categories (e.g. Vulnerable, Endangered and Critically Endangered).

### Relationship between λ -elasticity distributions and life history traits

**Figure 1:**
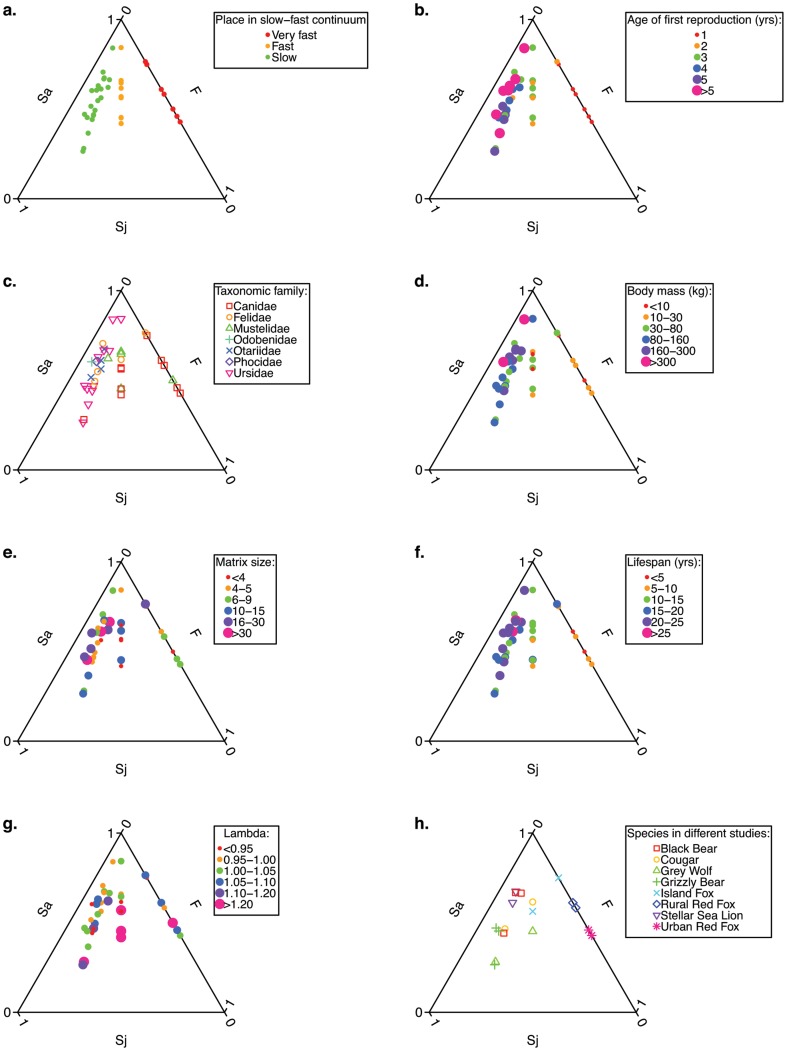
Triangular elasticity patterns in Carnivora species. Relation between elasticity patterns of Carnivora and (a) place in the slow-fast continuum, (b) age of first reproduction (c) taxonomic family, (d) average adult body mass, (e) matrix dimensions, (f) average life span, (g) projected population growth rate λ. Panel h shows different studies on the same species. Age of first reproduction was deduced from the matrix models. Body mass and life span were copied from the descriptions of the various authors, or, when missing, from various internet sources.

We plotted the *Sj*, *Sa* and *F* elasticity sums of each of the 38 matrix models in triangular elasticity graphs (Fig. 1). The foremost distinguishable pattern is the division of the data points in three distinct groups (Fig. 1a.). Many Carnivora are slow reproducers, which commonly have a low elasticity for fecundity. These animals are represented towards the left axis of the graph. There are also some very fast reproducing Carnivora species such as the red fox and the stoat. These animals start reproducing within their first year, so they do not have a juvenile stage. Therefore, their data points are located on the fecundity axis (at the right in Fig. 1a). Between these slow and very fast reproducing groups, a group of fast reproducing animals is visible: they start reproducing in their second year (Fig. 1b). Especially in age-based matrix models, age at first reproduction thus has a large impact on the contribution (i.e. elasticity value) of juvenile survival to the population growth rate. This can directly be seen in these models: age of first reproduction determines how many juvenile classes there are [22],[44]. In age-based matrix models, as determined by loop analysis, the summed elasticity value of juvenile survival is equal to the summed elasticity value of fecundity times the number of juvenile classes.

There was no relationship between the distribution of elasticity values and the taxonomic family (Fig. 1c). The elasticity of *F* seems to increase with smaller body mass (Fig. 1d) and smaller matrix sizes (Fig. 1e), because fecundity is usually more important in short-lived animals (Fig. 1f), for which smaller matrices tend to have been constructed. Too small matrices may have unrealistic biases towards reproduction loops, hereby artificially inflating *F* elasticities. The elasticity of *F* may also be increasing with increasing λ, as is generally the case [14], but this trend is only very weak in this dataset (Fig. 1g).

One way to investigate the role of the choice for a particular matrix model is to compare different studies of the same species. Different studies on the same Carnivora species give fairly similar results (Fig. 1h). For the wolf, cougar, island fox, black bear and grizzly bear, the studies differed in how the elasticity of survival was distributed over the juvenile and adult phases. Since for all of these species, the λ of the different populations did not differ much, the reason for these differences was likely the use of very different life cycle models. Of the wolf studies, one used an age-based life cycle with one non-breeding juvenile stage [45], while the other one used a stage-based life cycle with 6 stages, of which only the ‘dominants’ reproduce [28]. Both cougar studies used age-based life cycle models, but the difference in elasticity pattern was mostly caused by the number of juvenile classes, which was three in one study [46], and only one in the other [41]. The same goes for the grizzly bear studies, where the outlier study [47] has a much smaller number of adult classes then the other two studies [48],[49]. The data point of one of the island fox studies is located on the upper right axis because the matrix in this study [36] did not have a juvenile stage, while the other study had one [50]. Overall, the emerging picture is that species characteristics determine especially the elasticity partitioning in *F* and *S*, but that model characteristics can affect the partitioning in *S_j_* and *S_a_*.

### Demographic loop elasticities and the slow – fast continuum

**Figure 2:**
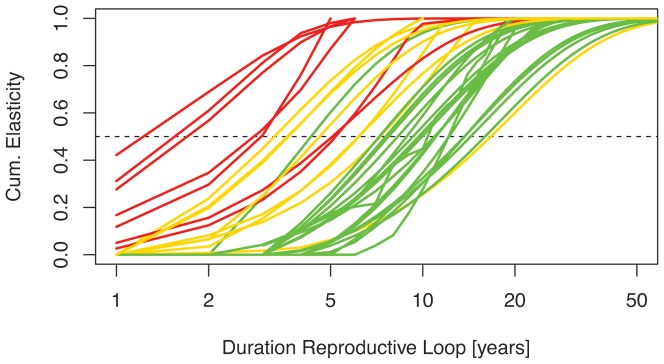
Cumulative elasticity of reproduction loops within age-based Leslie matrix models of Carnivora populations. Each line represents 1 study (see Table 1). Since the elasticity values of all life cycle loops add up to 1, the cumulative elasticity sum of loops of increasing length (i.e. increasing duration of the reproduction loops) reaches 1 at the maximal loop length of each matrix model. The red lines represent populations of ‘very fast’ species (see Fig. 1), yellow lines represent ‘fast’ species, and green lines ‘slow’ species. Three studies (walrus, polar bear, cheetah) are not plotted here because the life spans calculated from the matrix models were unrealistically long (see Appendix S1 for details and a plot including those three studies).

To overcome the effect of the abrupt transition from juvenile to adult stages on the elasticity sums of *S_j_* and *S_a_*, we also studied the elasticity patterns of the age-based matrices into which each of the studied matrices were converted. These age-based matrices only contain life cycle loops that include a reproduction event. Plotting the cumulative elasticity values of loops against their duration, a continuum along the slow-fast gradient emerged rather than discrete groups (Fig. 2). The ‘slow’, ‘fast’ and ‘very fast’ groups of Fig. 1 are distinct, but there is also some overlap between the groups. The two cougar studies are now displayed much closer together, with comparable age (6.2 and 7.6) at which 50% of the reproductive loop elasticity is reached (Table 1). Of the grizzly bear studies, the Pease and Mattson [47] study was now closer to the Wielgus et al. [49] study (both with 50% cumulative loop elasticity age of 10 years), than to the Wielgus [48] study (12 years).

**Figure 3:**
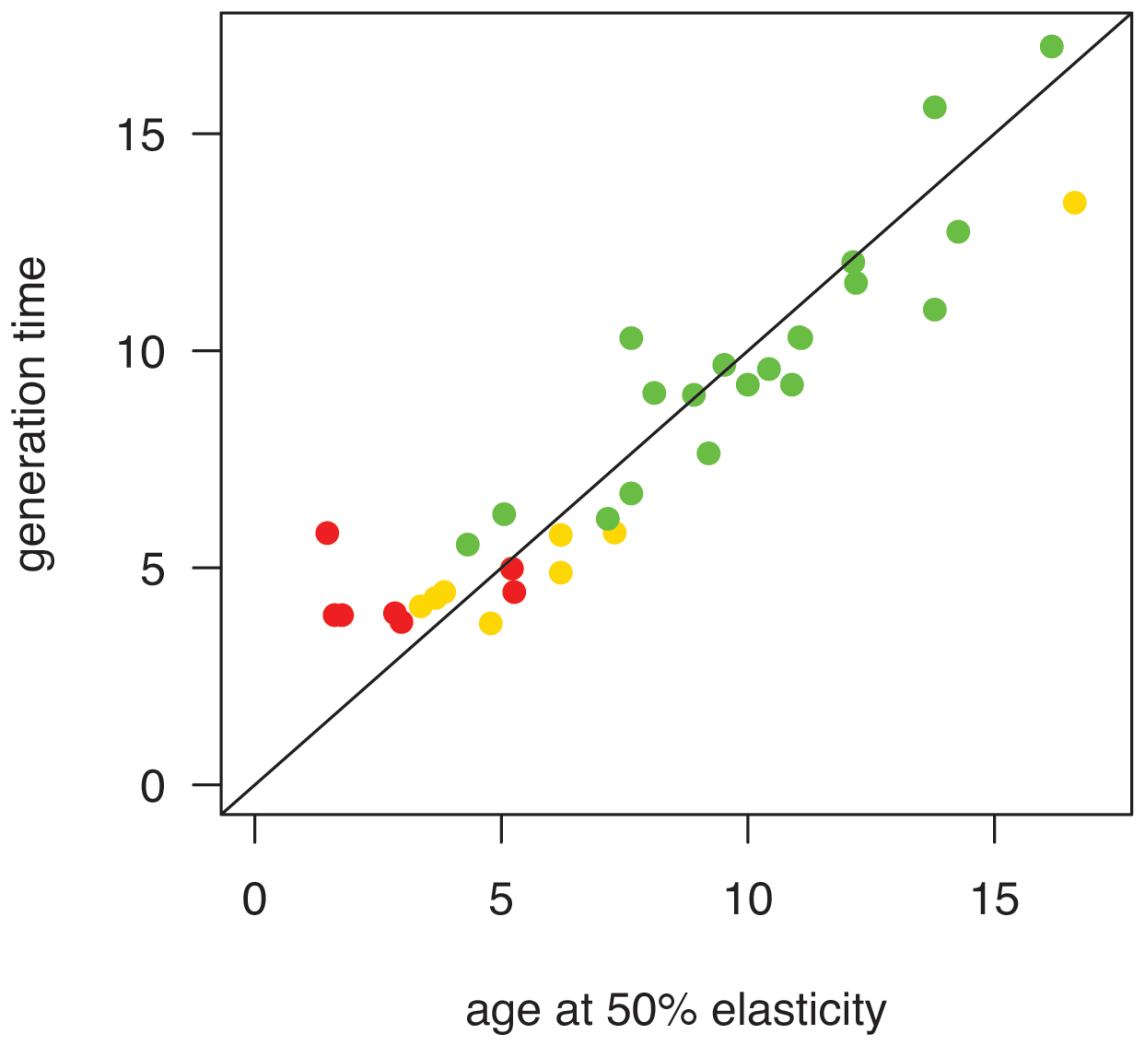
Relationship between the age of 50%-loop-elasticity and generation time, for multiple Carnivora matrix models. The 1:1 line indicates shows that these metrics, which are calculated from the same matrices, are closely related. Colors are the same as in Figures 1a and 2.

Variation between studies in the age of 50% cumulative loop elasticity can be understood as differences in generation time (see Table 1 and Appendix S1), since these two metrics were strongly correlated (r = 0.95, n = 37, p<0.001) and show a more or less 1∶1 relation (Fig. 3). Age of 50% cumulative loop elasticity was also strongly positively correlated with body mass (r = 0.94, p<0.001) and age of first reproduction (r = 0.66, p<0.001), positively correlated with the estimated life span (r = 0.48, p = 0.002), but not correlated with the projected population growth rate (r = −0.20, p = 0.23) or matrix dimension (r = 0.24, p = 0.15).

## Discussion

### Slow-fast continuum

The aim of our study was to investigate whether the range and patterns of life histories among Carnivora species are similar to those of other mammalian groups; and we show that they are very similar. Based on this similarity, extrapolation of elasticity patterns to other species is possible for species on which little research has been done and for which few demographic data are available. If information is available on key species characteristics, which we show to correlate with the elasticity distribution over the different components, then it should be possible to base certain predictions on this information. Species characteristics that showed a clear correlation with the elasticity distribution in this study were body mass and the reproduction speed (Figs. 1 and 2); the former suggesting that allometric relationships [51] also apply to population dynamics to some extent, while the latter being well characterized by the age at first reproduction (Fig. 1b and 2) and adult life span (Fig. 1f).

Along the slow-fast continuum, fast Carnivora generally have a higher elasticity for fecundity than slow Carnivora, which have a higher elasticity for survival. This is similar to results found for other animals, including birds [52], reptiles [53] and other mammals [22]. Based on the`se results, it is possible to estimate for a certain species how high its elasticity for fecundity or survival will be based on its place in the slow-fast continuum. Our results also show that caution should be taken when interpreting the partitioning of elasticity in juvenile survival and adult survival (as in Fig. 1 and [22]), since this depends on the chosen model structure: from what point onwards do individuals enter the ‘adult’ stage. We strictly defined adult stages to start with the first stage for which the reproduction rate is above zero, but this may have underestimated the average length of the juvenile phase. Furthermore, different authors tend to choose different model structures for the same species (Table 1, Fig. 1h). In addition to these modeling choices by the authors of the published matrix models, it needs to be kept in mind that these matrix models were based on vital rate values that were observed at natural population densities, while the vital rates were not explicitly modeled as a function of population density. This means that our comparison of elasticity values across species and studies is based on the assumption that meaningful patterns can be discerned when a large enough number of elasticity matrices is used, even though they are valid only for the encountered population densities and growth rates.

We have suggested a new method for showing how species are positioned along the slow-fast continuum by plotting cumulative loop elasticity against reproductive loop length, after transforming all matrices into age-based versions. Cumulative loop elasticities represent the demographic contributions of individuals of different generation length, as depicted by the close match between species generation time and 50% cumulative demographic loop elasticity (Fig. 3).

Demographic loop analysis not only overcomes the problem of the somewhat arbitrary definition of the onset of the adult phase, but also shows the range of important life cycle durations within a population. Variation in the length of reproduction loops with considerable elasticity (i.e. the ages of parents at which offspring production contributes to population growth) as depicted in figure 2 shows that the fast-slow continuum is not only apparent across species, but also within populations. Contributing reproduction ages vary considerably within and between individuals in all studied populations. Given the spread of reproductive loop durations among and within species, it is important to acknowledge the within-population variation in reproduction speed in population viability analyses. For the extrapolation to unstudied species it is good news, however, that the within-population variation in contributing reproduction loops scales nicely with the age of 50% cumulative loop elasticity (notice the logarithmic x-axis in Fig. 2).

### Similarities and differences with other mammals

Because Carnivora are not very well studied, it would be very useful if it were possible to compare unstudied species not only with other Carnivora, but also with mammals from other taxa. Heppell et al. [22] developed simple age-structured matrix models for 50 mammal populations, parameterized by juvenile survival, mean adult survival, age at maturity, and mean annual fertility. We found that the elasticity patterns of Carnivora populations are remarkably similar to patterns of the other mammals found by Heppell et al. [22]. This similarity occurs even though their specific place in the food chain means that population densities are low, causing them to be more vulnerable for known threats. Additionally, their position in the food chain as top or mesopredators makes it particularly complex to model their population dynamics. Nevertheless, Carnivora showed the same range (along the slow and fast continuum) of population dynamics as many other mammals. Our results suggest that this is a general pattern among mammals, driven by species life history structure, regardless of the specific taxon or position in the food chain.

### Rules of thumb for Carnivora conservation

It is highly desirable to be able to make predictions about the elasticity values of certain life cycle components and the responses of a species to different management strategies without first having to acquire large amounts of demographic data. For many endangered species, there is not enough time and money for a thorough study of population dynamics. Therefore, the clear slow-fast pattern in reproductive loop elasticity (Fig. 2) and triangular elasticity graphs (Fig. 1a) are encouraging, as well as their similarity to other mammals. Based on our and Heppell et al.'s [22] analyses, the population growth rate of slow species generally has a high elasticity for adult survival, while faster species tend to have a higher elasticity for fecundity. Conservation strategies should ideally be based on such population growth elasticity patterns. All else being equal (but see discussion below), the most effective management targets adult survival for slowly reproducing species, and fecundity for fast reproducers. This is well illustrated by one of the slowest species studied here, the black bear [54], with an elasticity of 0.66 for adult survival, and of 0.08 for fecundity. The authors recommended reducing adult female mortality by limiting road kill to conserve the population. For the short-lived stoat with an elasticity of 0.5 for fecundity, however, Wittmer et al. [42] suggested fertility control as a pest management strategy.

These generalizations suggest it should be possible to formulate ‘rules of thumb’ for the management strategies of threatened carnivore species for which data are limited or non-existing. This will be particularly useful for endangered species for which management cannot wait for long-term field studies to parameterize population models. For example, for a slow reproducer such as the critically endangered Hawaiian monk seal [1], conservation strategies targeted on adult survival are expected to be more effective. The major threats jeopardizing adult survival include food limitation due to competition with fisheries and entanglement in marine debris such as fishing nets and lines [55]. Conservation strategies focusing on eliminating entanglement in fishing nets together with habitat protection have been shown to be successful for the Hawaiian monk seal [56]. Recommendations for extensively studied sea turtles, suffering from many of the same problems and reproducing slowly as well, were very much the same [21]. It should be noted that also for slowly reproducing species reproduction should be successful for populations to increase, despite the low fecundity elasticities [14].

For a slow reproducer such as the jaguar (*Panthera onca*) conservation actions focusing on adult mortality reduction will be essential. Therefore, additionally to habitat protection, actions such as the development of wildlife passes along main roads can help to reduce adult mortality [57]. Additionally it will be important to establish other forms of corridors that ensure safe dispersal of adults and juveniles [58]. Furthermore it is not unlikely that adults are killed due to cattle predation; therefore the implementation of cattle insurance programs [59] could be essential in some regions to help reducing jaguar adult mortality. Previous studies have shown that it is possible to predict which areas will be more prompt to jaguar-human conflict [60], and the development of cattle insurance programs can thus be effectively targeted. Although the jaguar is the least known of the large felids, by knowing its place in the slow-fast continuum we can inform some conservation actions targeted to increase population growth.

On the other hand, for a fast reproducer such as the critically endangered Malabar civet [61], conservation actions towards fecundity are recommended. Malabar civet is an endemic to the Western Ghats of India, it is reported that its fecundity is reduced by lack of suitable mates due to habitat fragmentation and by high young mortality due to weeding at plantations [62]. However, given that the species' major threat is habitat loss and degradation and that the implementation of protected areas is unlikely due to high human population density in the region [61], the implementation of Conservation Breeding Programs may be a short-term solution to ensure population growth until habitat is identified or restored [62],[63].

We showed that Carnivora elasticities are similar to those of other mammals across the slow and fast continuum. This information is valuable, because it gives the possibility to estimate the expected elasticity distribution to inform preliminary conservation plans. Of course, other factors should also be considered. The population growth rate λ, for example, has been shown to shift the elasticity distribution towards higher elasticity for fecundity for growing population, and towards higher elasticity for survival for declining populations [14],[64]. At the same time annual population growth rates (and thus λ) are more variable in shorter-lived species. However, among the 38 studied matrix models λ and life spans were not significantly correlated (correlation coefficient = −0.22, P = 0.21), enabling a fair comparison along the slow-fast continuum in that respect.

What also needs to be considered when translating elasticity patterns to conservation management is that some management options are more costly than others, but also that some vital rates may be more prone to improvement than others; a survival rate will never be higher than one, and an animal can only produce so much offspring. It is not uncommon that vital rates with high elasticity have not much space for improvement, but much opportunity for decline: conservation actions should still target these rates [14]. If survival is fairly high and a population is in decline anyway, other vital rates need to be targeted as well.

The clear slow-fast continuum implies that even without thoroughly studying a species, it is possible to make tentative management plans for unstudied species, based on the species' body mass, age of first reproduction and/or life span, in order of decreasing value for prediction. Of course, the more of these species characteristics are known, the better the estimation of its position on the slow-fast continuum and the degree of within-population variation will be. Extrapolation of elasticity patterns thus is possible and especially useful for highly endangered species for which management cannot wait for long-term field studies to parameterize population models.

## Conclusions

Despite their specific place in the food chain, and despite some uncertainties in the models, our results suggest that the population dynamics and elasticity distributions of Carnivora are remarkably similar to those of other mammals and cover an equally wide range. The generality of the slow-fast continuum of elasticity values in mammals, and the correlations with simple information life body mass, age of first reproduction and life span, creates an opportunity to base tentative management plans of Carnivora on the population dynamics of similarly slow or fast well-studied mammals. Of course, such first management plan should be combined with demographic studies and an adaptive management program [65] in which direct responses to management and population modeling are used to fine-tune the management of specific populations.

## Supporting Information

Appendix S1 Loop Analyses.
(PDF)
