# Supplementary material for: Carnivora Population Dynamics Are as Slow and as Fast as Those of Other Mammals: Implications for Their Conservation
Source: PLoS One. 2013 Aug 12;8(8):e70354. doi: 10.1371/journal.pone.0070354 (PMC3741307; doi:10.1371/journal.pone.0070354)
Supplement: Appendix S1 — Loop Analyses. (PDF) [file pone.0070354.s001.pdf]

Appendix S1: Loop Analyses

Madelon van de Kerk, Hans de Kroon, Dalia A. Conde, Eelke Jongejans

July 17, 2013

Abstract

In this appendix to the paper 'Carnivora population dynamics are as slow and as fast as that of other mammals: implications for their conservation' we explain how we transformed age/stage-based matrices into purely age-based Leslie matrices.

The published matrix models reviewed in our paper use a variety of model structures. Some are purely age-based Leslie matrices (Leslie 1945), while others are also partly stage-based. Those stages can be either quality-based (e.g. dominants vs dispersers) or age-related (e.g. 'juveniles', or 'adults older than 5 years'). Such stage-based matrices often contain self-loops that do not contain a reproduction step in them (van Groenendael et al. 1994). The elasticity of those self loops are difficult to compare between studies. Therefore we decided to remove these self loops by transforming all matrices into purely age-based matrices that contain only reproduction loops. This allowed us to directly compare, between studies and species, the length and elasticity of life cycle loops (see Figures SS1 and SS2).

1 Making age-based matrices

Of the 38 analyzed matrices 19 had an absorbing "old age" class, as can be seen in the example of the leopard matrix (Balme et al. 2009):

$$\begin{pmatrix} 0 & 0 & 0 & 0.575 \\ 0.764 & 0 & 0 & 0 \\ 0 & 0.656 & 0 & 0 \\ 0 & 0 & 1 & 0.868 \end{pmatrix}$$

in which classes represent one-, two-, three-year old and older leopards. This can be transformed into a purely age-based matrix:

$$\begin{pmatrix} 0 & 0 & 0 & 0.575 & 0.575 & 0.575 \\ 0.764 & 0 & 0 & 0 & 0 & 0 \\ 0 & 0.656 & 0 & 0 & 0 & 0 \\ 0 & 0 & 1 & 0 & 0 & 0 \\ 0 & 0 & 0 & 0.868 & 0 & 0 \\ 0 & 0 & 0 & 0 & 0.868 & 0 \end{pmatrix}$$

Here all six-year olds die, which reduces the projected population growth rate (λ) from 1.090 to 0.945 (a 13% reduction). Using a larger matrix (20$\times$20) brings up (λ) closer to that of the original matrix (1.087; a 0.3% reduction). To fully represent the original model we extended the age-based matrix to a 100$\times$100 matrix, which has a λ indistinguishable from the original matrix. Note that, although this model has 100 age-classes, hardly any animals reach that age due to annual mortality. In the stable stage distribution only 1% of the animals is projected to be older than 20 years. These old ages might be biologically unrealistic, but directly a result of the self-loop in the bottom right corner of the original matrix.

Some matrices were more complex than the leopard model, however. For instance, in the case of the polar bear study by Hunter et al.

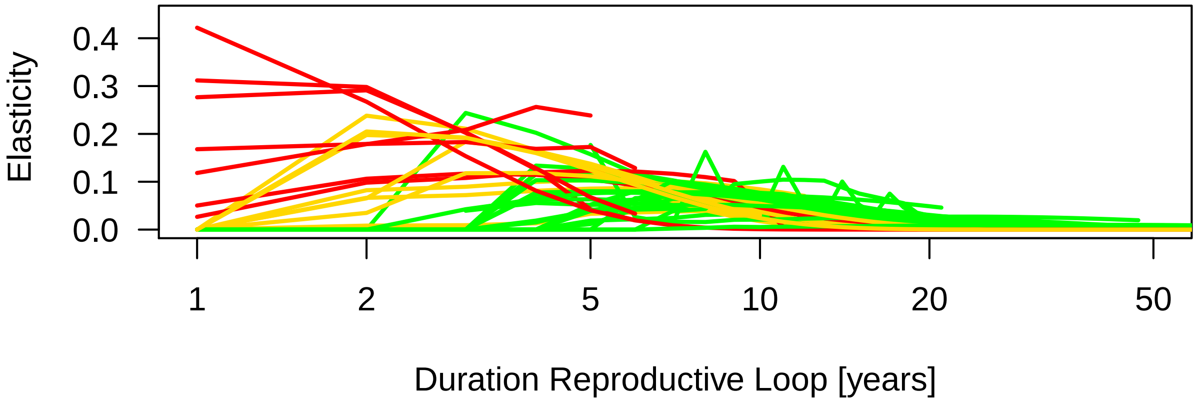

Figure S1: Elasticity of reproduction loops within age-based Leslie matrix models of Carnivora populations. Each line represents 1 study (see Table 1 in the main text), and loop elasticity values are plotted against the duration of the reproductive loops. Elasticity values are summed for loops of equal length. The red lines represent populations of 'very fast' species (see Fig. 1 in the main text), yellow lines represent 'fast' species, and green lines 'slow' species.

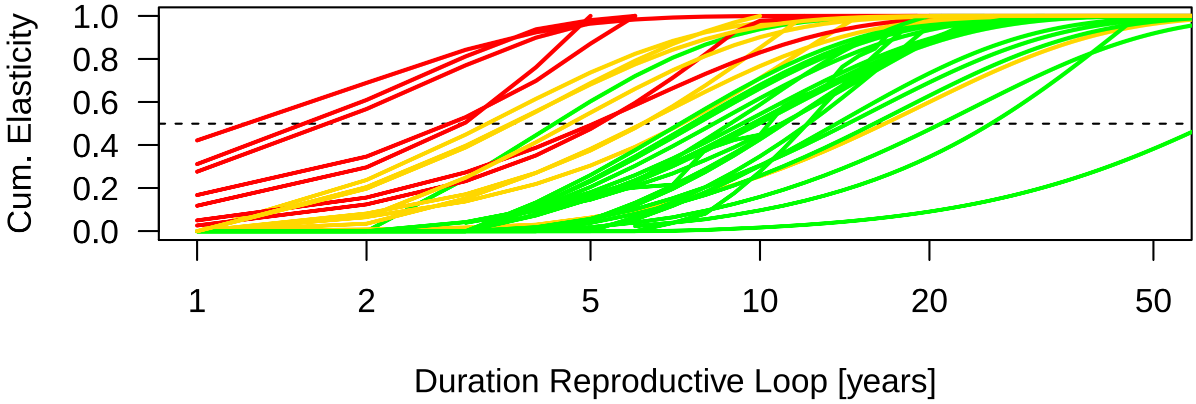

Figure S2: Cumulative elasticity of reproduction loops within age-based Leslie matrix models of Carnivora populations. Each line represents 1 study (see Table 1 in the main text). Since the elasticity values of all life cycle loops add up to 1, the cumulative elasticity sum of loops of increasing length (i.e. increasing duration of the reproduction loops) reaches 1 at the maximal loop length of each matrix model. The red lines represent populations of 'very fast' species (see Fig. 1 in the main text), yellow lines represent 'fast' species, and green lines 'slow' species.

(2010), surviving adults move between three stages: without young, with cub, and with yearling (as these stage influence next year's reproduction rates). To get rid of the non-reproductive loops between those adult stages, we simply added an age structure for these adult bears, with 3 stages for 5-year olds, 3 stages for 6-year olds, etc.

In all cases but two, the λ of our restructured matrix was within 0.0001 of that of the original matrix. Such small differences in λ have no discernible effects on the elasticity patterns studied in this paper. The first of the two exceptions in which the λ-difference was slightly larger was the giant panda matrix by Carter et al. (1999), which had a two-year time step. After converting this to a matrix model with a one-year time step λ was 0.96% lower. The second exception was the polar bear matrix, for which we constructed a 135×135 matrix (allowing a maximum age of 47 since from age 4 there are 3 different stages possible), of which the λ was 0.65% lower than that of the original, published matrix. Larger matrix dimensions would have reduced this difference further, but also increased computation time considerably for the loop elasticity algorithm (see below). However, these small differences hardly affected model dynamics.

2 Loop elasticity values

For Leslie matrices it is very easy to calculate the loop elasticity values. Given the elasticity matrix of e.g. the 6×6 leopard matrix

$$\begin{pmatrix} 0 & 0 & 0 & 0.073 & 0.067 & 0.062 \\ 0.202 & 0 & 0 & 0 & 0 & 0 \\ 0 & 0.202 & 0 & 0 & 0 & 0 \\ 0 & 0 & 0.202 & 0 & 0 & 0 \\ 0 & 0 & 0 & 0.129 & 0 & 0 \\ 0 & 0 & 0 & 0 & 0.062 & 0 \end{pmatrix}$$

we can see that the characteristic elasticity values of the three loops are in the top row (which contains the reproductive rates). Loop-characteristic elasticity values are the elasticity values of those matrix elements that only occur in a particular life cycle loop. To calculate the elasticity value of the entire loop we just have to multiply those characteristic elasticity values with the length of those loops (van Groenendael et al. 1994). In the case of Leslie matrices loop lengths are equal to the column number of the reproductive elements in the top row. The loop elasticity values are thus 0.073×4=0.293, 0.067×5=0.336 and 0.062×6=0.371. The sum of the elasticity values of all loops is 1. It are these values which are plotted for each species in figure S1 (using very large age-based matrices), while cumulative loop elasticity values are plotted in figure S2.

It is much harder (and sometimes impossible) to detect all characteristic loop elasticity values for more complex matrices like in the large age/stage-based matrix for the polar bear. In those cases we used an algorithm developed by Güneralp (2007), identifying and subtracting the shortest loops first from the elasticity matrix.

3 Loop elasticity patterns

To describe the cumulative loop elasticity patterns in figure SS2 we estimated the loop length (in years) at a cumulative loop elasticity of 0.5 (i.e. the age at which 50% of the contribution to λ has already occurred). We used the *approx* function in R (R Core Team 2012) for these estimations. This age of 50%-loop-elasticity is strongly correlated with the matrix metric 'generation time', which we calculated using the R package *popbio* (Stubben & Milligan 2007). Generation time is defined here as the $\log(R_0)/\log(\lambda)$ ratio. The 50%-loop-elasticity, λ, R_0 and generation time are listed for all matrices in table S1.

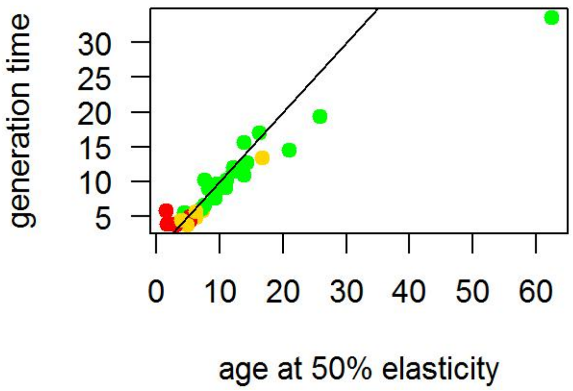

Figure S3: Relationship between the age of 50%-loop-elasticity and generation time, for multiple Carnivora matrix models. The 1:1 line indicates shows that these metrics, which are calculated from the same matrices, are closely related. Colors are the same as in figures S1 and S2

Figure SS3 shows that the 50%-loop-elasticity age and generation time are related more or less 1:1, though for 'slower' Carnivora generation time is lower than the 50%-loop-elasticity age (most notably for the walrus model by Gilbert & Udevitz (1997)). This might partly be because both generation time and elasticity functions have λ (or $\log(\lambda)$) in the denominator. However, these metrics can vary independently as well. Closer inspection reveals that the ratio of 50%-loop-elasticity and generation time can be statistically explained by a combination of 3 variables: λ, dimension of the original matrix, and the non-reproductive self-loop elasticity of the original matrix ($R^2 = 0.54$ in a simple linear regression; $p<0.001$ for all 3 variables). This probably means that stage-based matrices of which λ are low, matrix dimension are large and non-reproductive self-loop elasticity are high, cannot be realistically converted to Leslie matrices, probably because the survival of the older classes cannot be properly estimated from the data. This is the case in the walrus model (λ = 0.98, dimension original matrix = 26, non-reproductive self-loop elasticity = 0.51), polar bear model (0.99, 6 and 0.76, respectively) and cheetah model (0.96, 8 and 0.55, respectively). In these cases where populations are projected to slightly decline and adult survival self-loops contribute more than 50% to population growth, the mass of the loop elasticity in our purely Leslie matrices are pulled towards longer lengths than might be expected from generation times. We decided to remove these 3 cases from Figures 2 and 3 in the main text, because the loop elasticity patterns might suggest much higher reproductive ages than normal for these species. Figures SS1 and SS2 of this appendix, however, do show results from all 38 matrix models.

References

Bakker, V. J., Doak, D. F., Roemer, G. W., Garcelon, D. K., Coonan, T. J., Morrison, S. A., Lynch, C., Ralls, K. & Shaw, R. (2009), 'Incorporating ecological drivers and uncertainty into a demographic population viability analysis for the island fox', *Ecological Monographs* **79**, 77–108.

Balme, G. A., Slotow, R. & Hunter, L. T. B. (2009), 'Impact of conservation interventions on the dynamics and persistence of a persecuted leopard (Panthera pardus) population', *Biological Conservation* **142**, 2681–2690.

Carter, J., Ackleh, A. S., Leonard, B. P. & Wang, H. (1999), 'Giant panda (Ailuropoda melanoleuca) population dynamics and bamboo (subfamily Bambusoideae) life history: a structured population approach to examining carrying capacity when the prey are semelparous', *Ecological Modelling* **123**, 207–223.

Chapron, G., Quenette, P.-Y., Legendre, S. & Clobert, J. (2003), 'Which future for the French Pyrenean brown bear (Ursus arctos) population? An approach using stage-structured deterministic and stochastic models', *Comptes Rendus Biologies* **326**, 174–182.

Crooks, K. R., Sanjayan, M. A. & Doak, D. F. (1998), 'New insights on cheetah conservation through demographic modeling', *Conservation Biology* **12**, 889–895.

Cross, P. C. & Beissinger, S. R. (2001), 'Using logistic regression to analyze the sensitivity of PVA models: a comparison of methods based on African wild dog models', *Conservation Biology* **15**, 1335–1346.

Freedman, A. H., Portier, K. M. & Sunquist, M. E. (2003), 'Life history analysis for black bears (Ursus americanus) in a changing demographic landscape', *Ecological Modelling* **167**, 47–64.

Gerber, L. R., Tinker, M. T., Doak, D. F., Estes, J. A. & Jessup, D. A. (2004), 'Mortality Sensitivity in Life-Stage Simulation Analysis: a Case Study of Southern Sea Otters', *Ecological Applications* **14**, 1554–1565.

Gilbert, J. R. & Udevitz, M. S. (1997), 'Adaptation of a stage-projection model for species with multiple year reproductive cycles', *Ecological Modelling* **97**, 47–57.

Gorman, T. A., McMillan, B. R., Erb, J. D., Deperno, C. S. & Martin, D. J. (2008), 'Survival and cause-specific mortality of a protected population of river otters In Minnesota', *American Midland Naturalist* **159**, 98–109.

Güneralp, B. (2007), 'An improved formal approach to demographic loop analysis', *Ecology* **88**, 2124–31.

Haines, A. M., Tewes, M. E., Laack, L. L., Horne, J. S. & Young, J. H. (2006), 'A habitat-based population viability analysis for ocelots (Leopardus pardalis) in the United States', *Biological Conservation* **132**, 424–436.

Harwood, J. (1978), 'The effect of management policies on the stability and resilience of British grey seal populations', *Journal of Applied Ecology* **15**, 413–421.

Hebblewhite, M. (2003), 'Black bear (Ursus americanus) survival and demography in the Bow Valley of Banff National Park, Alberta', *Biological Conservation* **112**, 415–425.

Holmes, E. E. & York, A. E. (2003), 'Using Age Structure to Detect Impacts on Threatened Populations: a Case Study with Steller Sea Lions', *Conservation Biology* **17**, 1794–1806.

Hostetler, J. A., Onorato, D. P., Jansen, D. & Oli, M. K. (2013), 'A cat's tale: the impact of genetic restoration on Florida panther population dynamics and persistence', *Journal of Animal Ecology* **82**, 608–620.

Hostetler, J. A., Walter McCown, J., Garrison, E. P., Neils, A. M., Barrett, M. A., Sunquist, M. E., Simek, S. L. & Oli, M. K. (2009), 'Demographic consequences of anthropogenic influences: Florida black bears in north-central Florida', *Biological Conservation* **142**, 2456–2463.

Hudgens, B. R. & Garcelon, D. K. (2011), 'Induced changes in island fox (Urocyon littoralis) activity do not mitigate the extinction threat posed by a novel predator.', *Oecologia* **165**, 699–705.

Hunter, C. M., Caswell, H., Runge, M. C., Regehr, E. V., Amstrup, S. C. & Stirling, I. (2010), 'Climate change threatens polar bear populations: a stochastic demographic analysis', *Ecology* **91**, 2883–2897.

Kohira, M., Okada, H., Nakanishi, M. & Yamanaka, M. (2009), 'Modeling the effects of human-caused mortality on the brown bear population on the Shiretoko Peninsula, Hokkaido, Japan', *Ursus* **20**, 12–21.

Lalas, C. & Bradshaw, C. J. A. (2003), 'Expectations for population growth at new breeding locations for the vulnerable New Zealand sea lion (Phocarctos hookeri) using a simulation model', *Biological Conservation* **114**, 67–78.

Lambert, C. M. S., Wielgus, R. B., Robinson, H. S., Katnik, D. D., Cruickshank, H. S., Clarke, R. & Almack, J. (2006), 'Cougar population dynamics and viability in the Pacific Northwest', *Journal of Wildlife Management* **70**, 246–254.

Leslie, P. H. (1945), 'On the use of matrices in certain population mathematics', *Biometrika* **33**, 183–212.

Macdonald, D. W. & Newman, C. (2006), 'Population dynamics of badgers (Meles meles) in Oxfordshire, U.K.: numbers, density and cohort life histories, and a possible role of climate change in population growth', *Journal of Zoology* **256**, 121–138.

McLeod, S. R. & Saunders, G. R. (2001), 'Improving management strategies for the red fox by using projection matrix analysis', *Wildlife Research* **28**, 333–340.

Miller, D. H., Jensen, A. L. & Hammill, J. H. (2002), 'Density dependent matrix model for gray wolf population projection', *Ecological Modelling* **151**, 271–278.

Mitchell, M. S., Pacifici, L. B., Grand, J. B. & Powell, R. A. (2009), 'Contributions of vital rates to growth of a protected population of American black bears', *Ursus* **20**, 77–84.

Novaro, A. J., Funes, M. C. & Walker, R. S. (2005), 'An empirical test of source-sink dynamics induced by hunting', *Journal of Applied Ecology* **42**, 910–920.

Pascual, M. A. & Adkison, M. D. (1994), 'The decline of the steller sea Lion in the Northeast Pacific: demography, harvest or environment?', *Ecological Applications* **4**, 393–403.

Pease, C. M. & Mattson, D. J. (1999), 'Demography of the Yellowstone grizzly bears', *Ecology* **80**, 957–975.

R Core Team (2012), 'R: A language and environment for statistical computing'.
URL: *http://www.r-project.org/*

Robinson, H. S., Wielgus, R. B., Cooley, H. S. & Cooley, S. W. (2008), 'Sink populations in carnivore management: cougar demography and immigration in a hunted population.', *Ecological Applications* **18**, 1028–1037.

Seignobosc, M., Hemerik, L. & Koelewijn, H. P. (2011), 'A demo-genetic analysis of a small reintroduced carnivore population: the otter (Lutra lutra) in the Netherlands', *International Journal of Ecology* **2011**, 1–11.

Stubben, C. & Milligan, B. (2007), 'Estimating and analyzing demographic models using the popbio package in R', *Journal of Statistical Software* **22**(11).

van Groenendael, J., de Kroon, H., Kalisz, S. & Tuljapurkar, S. (1994), 'Loop analysis: evaluating life history pathways in population projection matrices', *Ecology* **75**, 2410–2415.

Wielgus, J., Gonzalez-Suarez, M., Aurioles-Gamboa, D. & Gerber, L. R. (2008), 'A non-invasive demographic assessment of sea lions based on stage-specific abundances', *Ecological Applications* **18**, 1287–1296.

Wielgus, R. B. (2002), 'Minimum viable population and reserve sizes for naturally regulated grizzly bears in British Columbia', *Biological Conservation* **106**, 381–388.

Wielgus, R. B., Sarrazin, F., Ferriere, R. & Clobert, J. (2001), 'Estimating effects of adult male mortality on grizzly bear population growth and persistence using matrix models', *Biological Conservation* **98**, 293–303.

Wittmer, H. U., Powell, R. A. & King, C. M. (2007), 'Understanding contributions of cohort effects to growth rates of fluctuating populations', *Journal of Animal Ecology* **76**, 946–956.

Table S1: Studies from which Carnivora matrix models where derived (in the same order as in Table 1 in the main text), with information about the original and newly constructed age-based matrices. Dim1 and Dim2 are the dimension of the original and new age-based matrices. λ is the dominant eigenvalue of the new matrices. The ages at which 50% of the loop elasticity has already occurred are given, as well as the net reproductive rate (R_0) and generation time ($\log(R_0)/\log(\lambda)$ calculated from the new matrices.

Species & Study	Dim1	Dim2	λ	R_0	age50	genTime
Grey wolf (Chapron et al. 2003)	6	40	1.33	6.05	5.06	6.24
Grey wolf (Miller et al. 2002)	10	10	1.35	3.59	3.67	4.30
African wild dog (Cross & Beissinger 2001)	3	100	1.29	4.01	4.32	5.53
Culpeo fox (Novaro et al. 2005)	3	100	1.29	2.83	3.37	4.12
Island fox (Bakker et al. 2009)	2	100	0.87	0.54	5.26	4.44
Island fox (Hudgens & Garcelon 2011)	3	100	0.64	0.19	4.79	3.72
Red fox (Urban 1) (McLeod & Saunders 2001)	6	6	1.03	1.14	1.62	3.91
Red fox (Urban 2) (McLeod & Saunders 2001)	6	6	1.08	1.36	1.77	3.91
Red fox (Rural 1) (McLeod & Saunders 2001)	5	5	1.06	1.26	2.97	3.76
Red fox (Rural 2) (McLeod & Saunders 2001)	6	6	0.95	0.82	2.84	3.95
Cheetah (Crooks et al. 1998)	8	100	0.96	0.51	20.89	14.56
Ocelot (Haines et al. 2006)	4	100	1.05	1.60	9.53	9.68
Leopard (Balme et al. 2009)	4	100	1.09	2.18	8.11	9.03
Cougar (Lambert et al. 2006)	12	12	0.92	0.67	6.21	4.89
Cougar (Robinson et al. 2008)	6	100	0.88	0.42	7.63	6.71
Florida panther (Hostetler et al. 2013)	19	19	1.06	1.34	5.22	4.99
Eurasian otter (Seignobosc et al. 2011)	2	100	1.26	2.78	3.84	4.44
Sea otter (Gerber et al. 2004)	20	20	1.13	2.91	8.91	8.99
River otter (Gorman et al. 2008)	3	100	1.02	1.09	6.21	5.76
Badger (Macdonald & Newman 2006)	15	15	0.99	0.95	7.30	5.81
Stoat (Wittmer et al. 2007)	3	100	1.26	3.80	1.47	5.80
Walrus (Gilbert & Udevitz 1997)	26	300	0.98	0.44	62.52	33.57
Stellar sea lion (Holmes & York 2003)	32	32	1.00	1.00	11.09	10.29
Stellar sea lion (Pascual & Adkison 1994)	14	100	1.01	1.19	14.26	12.74
New zealand sea lion (Lalas & Bradshaw 2003)	26	26	1.00	0.99	11.05	10.32
California sea lion (Wielgus et al. 2008)	3	100	0.95	0.68	9.20	7.64
Grey seal (Harwood 1978)	7	100	1.08	3.17	13.79	15.61
Giant panda (Carter et al. 1999)	13	25	1.01	1.01	12.14	12.04
Black bear (Freedman et al. 2003)	69	69	1.02	1.19	10.89	9.22
Black bear (Hebblewhite 2003)	5	100	0.95	0.59	13.79	10.95
Black bear (Mitchell et al. 2009)	4	100	0.78	0.22	7.16	6.13
Florida black bear (Hostetler et al. 2009)	5	100	1.01	1.18	16.63	13.42
Eurasian brown bear (Chapron et al. 2003)	4	100	1.19	5.84	7.63	10.29
Grizzly bear (Wielgus et al. 2001)	50	50	1.05	1.61	10.43	9.57
Grizzly bear (Pease & Mattson 1999)	10	100	1.01	1.13	10.00	9.22
Grizzly bear (Wielgus 2002)	21	21	1.03	1.34	12.20	11.56
Japanese brown bear (Kohira et al. 2009)	5	100	1.06	2.61	16.16	17.01
Polar bear (Hunter et al. 2010)	6	135	0.99	0.82	25.81	19.44
